# Draft Genome Sequence of the Filamentous Fungus *Hypoxylon pulicicidum* ATCC 74245

**DOI:** 10.1128/genomeA.01380-17

**Published:** 2018-01-11

**Authors:** Matthew J. Nicholson, Kyle C. Van de Bittner, Arvina Ram, Leyla Y. Bustamante, Barry Scott, Emily J. Parker

**Affiliations:** a Ferrier Research Institute, Victoria University of Wellington, Wellington, New Zealand; b Biomolecular Interaction Centre, Department of Chemistry, University of Canterbury, Christchurch, New Zealand; c Institute of Fundamental Sciences, Massey University, Palmerston North, New Zealand

## Abstract

*Hypoxylon pulicicidum* strain MF5954 (ATCC 74245) (formerly classified as *Nodulisporium* sp.) is a filamentous fungal species known for its production of the secondary metabolite nodulisporic acid A. We present here the 41.5-Mb draft genome sequence for this organism.

## GENOME ANNOUNCEMENT

*Hypoxylon pulicicidum* strain MF5954 (ATCC 74245) is an endophytic fungus first isolated from a woody plant (*Bontia daphnoides*) in Hawaii, USA (1), and originally identified as *Nodulisporium* sp. (2, 3). *H. pulicicidum* is known for its production of nodulisporic acids, a group of bioactive indole diterpene secondary metabolites derived from emindole SB (4). Nodulisporic acid A is of particular significance because it exhibits highly potent insecticidal activity against blood-feeding arthropods while exhibiting no observable adverse effects on mammals (5, 6). Nodulisporic acid A exerts its action via activation of glutamate-gated chloride channels found in insects (7). The difficulties in achieving nodulisporic acid biosynthesis from *H. pulicicidum* and failure to achieve total chemical synthesis have limited the supply of this potentially beneficial anti-insect compound. The genome sequence of *H. pulicicidum* was sequenced to investigate the genes required for biosynthesis of nodulisporic acids in order to facilitate heterologous biosynthesis of these compounds.

*H. pulicicidum* genomic DNA was prepared by phenol-chloroform extraction (8) and treated with RNase A. One-third of a run with 300-bp paired-end fragment reads was done on an Illumina MiSeq instrument by New Zealand Genomics Limited (NZGL) and attained approximately 49-fold genome coverage. Reads were dynamically trimmed using the SolexaQA++ package to their longest fragment such that base call error rates did not exceed *P* = 0.01 and the minimum length of paired-end reads was 25 bp. *De novo* assembly was performed using SPAdes (version 3.5.0) with the default parameters, using a kmer range of 39 to 127, and scaffolding was performed using SSPACE version 1.10 and GapFiller version 3.0. The final assembly consisted of 204 contigs over 500 bp with an average length of 203,162 nucleotides. The total number of nucleotide residues was 41,444,948, with a GC content of 45.87%. The largest contig was 3,773,335 bp, the *N*_50_ was 580,679 bp, and the *L*_50_ was 17.

Bioinformatic analyses, including BLAST and FGENESH gene-finding software (9), were used to identify a gene cluster that is responsible for nodulisporic acid biosynthesis (10). The annotated nucleotide sequence of the *H. pulicicidum* nodulisporic acid gene cluster has been deposited at DDBJ/ENA/GenBank with accession number MG182145.

### Accession number(s).

This whole-genome shotgun project has been deposited at DDBJ/ENA/GenBank under the accession number PDUJ00000000. The version described in this paper is version PDUJ01000000.
